# Association between obesity and cervical intraepithelial neoplasia: results from a case control study in south western Uganda

**DOI:** 10.1186/s12905-023-02315-1

**Published:** 2023-04-04

**Authors:** Frank Ssedyabane, Joseph Ngonzi, Rogers Kajabwangu, Josephine Nambi Najjuma, Deusdedit Tusubira, Thomas C Randall

**Affiliations:** 1 Department of Medical Laboratory Science, Faculty of Medicine, Mbarara University of Science of Science and Technology, P.O. Box 1410, Mbarara, Uganda; 2 Department of Obstetrics and Gynaecology, Faculty of Medicine, Mbarara University of Science of Science and Technology, P.O. Box 1410, Mbarara, Uganda; 3 Department of Biochemistry, Mbarara University of Science of Science and Technology, P.O. Box 1410, Mbarara, Uganda; 4 Department of Nursing, Mbarara University of Science of Science and Technology, P.O. Box 1410, Mbarara, Uganda; 5 Massachusetts General Hospital, Boston, USA; 6 Mbarara Regional Referral Hospital, P.O. Box 40, Mbarara, Uganda

**Keywords:** Cervical intraepithelial neoplasia, Obesity, Body Mass Index, Cervical precancer

## Abstract

**Background:**

Though obesity has been said to be associated with a number of malignancies including cervical cancer, its association with cervical intraepithelial neoplasia (CIN) is still a contentious issue. This study was designed to determining the prevalence and association between obesity and CIN.

**Methods:**

This was an unmatched case control study, involving women with cervical intraepithelial neoplasia (cases) and those negative for intraepithelial lesions or malignancy (controls) at the cervical cancer clinic of Mbarara Regional Referral Hospital, in south-western Uganda, between April and November 2022. Cases and controls provided written informed consent and were recruited in a ratio of 1:1. Cases were identified by visual inspection with acetic acid (VIA) and subsequent confirmation with cytology and/or histology. Demographic information was collected using an enrolment form and height, weight and waist circumference were recorded. We calculated body mass index (BMI) and identified obese women as those with body mass index of ≥ 30 kg/m^2^ from both case and control groups. Central obesity was defined as waist: height ration of ≥ 0.5. Data was analysed using STATA version 17. Categorical variables were analysed using proportions, chi-square and logistic regression analysis to determine association between obesity and CIN. Our level of statistical significance was set at ≤ 0.05.

**Results:**

The prevalence of general and central obesity among cases was 25.5% (24/94) and 0% (0/94) respectively while the prevalence of general and central obesity among controls was 33.3% (37/111) and 0% (0/111) respectively. There was an increased prevalence of general obesity among women with low grade squamous intraepithelial lesions (LSIL). However, there was no statistically significant association between general obesity and CIN. Factors associated with general obesity included residing in Mbarara city (AOR 2.156, 95%CI 1.085–4.282, P-value 0.028), age group of 31–45 years (AOR 2.421, 95%CI 1.577–9.705, P-value 0.003) and ≥ 46 years (AOR 1.971, 95%CI 1.022–11.157, P-value 0.046).

**Conclusion:**

We observed an increased prevalence of general obesity among women with LSIL. However, there was no association between obesity and CIN. Factors associated with general obesity included residing in Mbarara city, and being in the age groups of 31–40 and ≥ 46 years. This highlights the need to rethink management of CIN to control other non-communicable diseases that could arise due to general obesity.

## Background

World over, there were 770,828 incident cervical cancer cases in 2020 [1]. Cervical cancer is ranked the second most common cancer among women of reproductive age [2] and it accounts for more than 270 000 deaths annually, mostly in developing countries [3] and most especially in sub Saharan Africa [4, 5]. In the East African region, Cervical Cancer stands at 43/100,000 cancer cases [6], and over 4,000 new cases are recorded annually in Uganda [7]. A well-proven way to prevent cervical cancer is to screen and detect pre-cancers (CIN) before progressing to invasive cancer [3]. Other methods of cervical cancer prevention include vaccination for girls aged 9 to 14 years, health education for both boys and girls as well as treatment for all that need to be treated [1, 8, 9]. Vaccination as well as other factors has also been reported to be synergic to treatment [10] and it has an impact on the risk of developing persistence and recurrence of cervical dysplasia after treatment [11–13].

Cervical intraepithelial neoplasia (CIN) represents a precancerous stage that develops before cervical cancer [14, 15] and is mainly a result of infection with High Risk Human Papilloma Virus (HRHPV) [16]. Preventing CIN is still the best approach to eliminating the cervical cancer burden [17, 18].

It is important to understand other risk factors to CIN as this may lead to more targeted primary prevention strategies to address the current cervical cancer burden [14]. There are secondary factors that lead to HPV infection, its persistence and development of precancerous lesions [19].

An increased BMI has been associated with a number of disease conditions ranging from cardiovascular diseases (CVD) to cancers [20]. Obesity has been thought to increase one’s risk of developing cervical cancer [21]. It is hypothesised that increased BMI tends to impair one’s immune system, leading to persistent HPV infections, leading to cervical cancer [22–24]. The odds of having CIN are said to be higher for those women with increased BMI [14]. Obesity has previously been shown to be associated with increased cervical cancer risk [25–29]. However, in a broader picture, the association between BMI and CIN is not well understood [21, 30, 31]. This study was therefore aimed at determining if there is an association between obesity and cervical intraepithelial neoplasia.

## Materials and methods

### Study setting

The study was carried out among women attending the cervical cancer clinic of Mbarara Regional Referral Hospital. This clinic screens an average of 15 mothers per day and operates five days a week. It serves 13 districts of south western Uganda. The confirmatory tests for cervical intraepithelial lesions, PAP smears or histology are performed in the pathology department of the hospital/university.

### Study design

This was an unmatched case control study. It included all women who sought cervical cancer screening services at the cervical cancer clinic of Mbarara Regional Referral Hospital. The cases were those women with a positive VIA and confirmed, with Pap smear cytology or histology, to have CIN while controls were those women with a negative VIA.

### Sampling procedure

Cases and controls were selected prospectively. Cases were recruited using purposive sampling. Then we employed the incidence density sampling method to obtain controls i.e. we recruited a control each time a case was identified.

### Sample size determination

The sample size was calculated using an online software, OpenEpi, Version 3, Open source calculator-SSCC. OpenEpi - Sample Size for Unmatched Case-Control Studies.

For this calculation, we considered a two-sided confidence level (1-alpha) of 95, a study power of 80%, a case to control ratio of 1, a proportion of cases with obesity as a component of metabolic syndrome of 40.99% [32] and a proportion of controls with obesity as a component of metabolic syndrome of 18.8% [33]. We also used the least extreme odds ratio of 3.0. This gave a minimum of 75 cases and 75 controls, considering module of Fleiss with continuity correction [34].

### Data collection

#### Demographic data collection

Demographic data was collected using a pretested enrolment form which was administered by research assistants, the cervical cancer clinic midwives. Participants were explained to and taken through the consent process, filling the enrolment form and anthropometric measurements. The study PI performed random daily checks on the procedures to ensure conformity to the study requirements and validity as well as completeness of the data. Data collected included age, region of residence, age at first sexual debut, family planning usage, HIV status, CD4 count, HIV viral load, highest level of education, number of life-time sexual partners, marital status, history of blood pressure and history of diabetes.

#### Body Mass Index and anthropometric measurements

Measurements of height (in meters) and weight (in kilograms) were taken using calibrated stadiometer and weighing scale respectively. We also measured waist circumference (in meters). We calculated BMI as the weight in kilograms divided by the square of the height in meters. We then categorized BMI as non-obese (< 30 kg/m2) or obese (30 ≤ kg/m2) in accordance with standard cut off points by the World Health Organisation as used in previous studies [35, 36]. We defined central obesity using the World Health Organisation categorisation, as a waist to height ratio of ≥ 0.5 [35, 36].

#### Data management and analysis

Data was collected by the research assistants and the Principal Investigator. It was then entered into Microsoft Excel (Ms 2007), computer database, imported and analysed using STATA 17 software. Descriptive statistics were used to characterize the population using frequencies, means ± standard deviations (SDs) using the Ind. t test. Chi-square, Fisher’s exact test and logistic regression analysis were used to describe categorical variables and assess associations between obesity and categorical variables. Univariate analysis was done first and we purposively selected variables, with p-values < 0.1, for multivariate analysis. A P-value of ≤ 0.05 was considered to be statistically significant at multivariate analysis.

#### Eligibility criteria

We included all women of 21 years and above, who reported to the cervical cancer clinic of Mbarara Regional Referral Hospital during the time of the study, screened positive with VIA or HPV DNA, consented to participate in the study, and later confirmed to have a precancerous lesion (CIN) by either Pap smear or histology. Any woman that was moribund (too ill) to consent was excluded.

## Results

We recruited a total of 188 women (94 controls and 94 cases), following our inclusion criteria. There were 36/94 controls with general obesity and 24/94 cases with general obesity. This resulted in the prevalence of general obesity among controls and cases of 38% and 26% respectively. Results from demographics show non-significant differences between cases and controls. However, mean age of participants, contraceptive used and highest level of education varied significantly across cases and controls. Table 1.

**Table 1:** Comparison of obesity and other demographic characteristics between women with and those without Cervical Intraepithelial Neoplasia

Variable		NO CIN (Controls)	CIN (Cases)	Test	p-value
		N = 94	N = 94		
Age		38.77 (± 8.27)	34.53 (± 7.79)	Ind. t test	< 0.001
Age group	19–30	20 (21%)	29 (31%)	Chi-square	0.061
	31–45	53 (56%)	55 (59%)		
	> 46	21 (22%)	10 (11%)		
History of high BP	No	71 (76%)	74 (79%)	Chi-square	0.6
	Yes	23 (24%)	20 (21%)		
History of Diabetes	No	80 (85%)	82 (87%)	Chi-square	0.67
	Yes	14 (15%)	12 (13%)		
Marital status	Single	14 (15%)	21 (22%)	Chi-square	0.43
	Married	60 (65%)	54 (57%)		
	Divorced	19 (20%)	19 (20%)		
Highest level of education	Never studied	13 (14%)	4 (4%)	Fisher’s exact	0.011
	Preprimary	5 (5%)	3 (3%)		
	Primary	35 (38%)	45 (48%)		
	Secondary	34 (37%)	25 (27%)		
	Tertiary	5 (5%)	9 (10%)		
	University	1 (1%)	8 (9%)		
HIV status	Negative	52 (55%)	50 (53%)	Fisher’s exact	0.77
	Positive	41 (44%)	44 (47%)		
	Unknown	1 (1%)	0 (0%)		
HIV Viral Load (copies/ml)		40.84 (± 161.33)	18.98 (± 85.10)	Ind. t test	0.4
Body Mass Index		28.00 (± ×/1.25)	28.00 (± ×/1.47)	Ind. t test, logged	1
CD4 count (cells/UL)		555.75 (± 362.32)	488.18 (± 366.428)	Ind. t test	0.7
Smoking	No	93 (99%)	86 (92%)	Fisher’s exact	0.035
	Yes	1 (1%)	7 (8%)		
Contraceptive use	No	55 (60%)	37 (41%)	Chi-square	0.01
	Yes	37 (40%)	54 (59%)		
Age at sexual debut		18.37 (± 3.04)	19.26 (± 3.50)	Ind. t test	0.065
Region of residence	Mbarara city	92 (98%)	91 (97%)	Fisher’s exact	1
	Rest of western Uganda	2 (2%)	2 (2%)		
	Central Uganda	0 (0%)	1 (1%)		
General obesity	Non obese	58 (62%)	70 (74%)	Chi-square	0.060
	Obese	36 (38%)	24 (26%)		
Central obesity	Non obese	94 (100%)	94 (100%)	Fisher’s exact	1.00
	Obese	0 (0%)	0 (0%)		

### Distribution of participant demographics with general obesity

The primary exposure of this study was obesity among participants with or without CIN. From all study participants, 60 participants had obesity while 128 were non obese. We observed a statistically significant difference in the distributions of age groups, HIV viral load, contraceptive use and age at sexual debut across obesity. Table 2.

**Table 2:** Distribution of participant demographics based on general obesity

Variable		No general obesity	General obesity	Test	p-value
		N = 128	N = 60		
Age		35.98 (± 8.56)	38.07 (± 7.60)	Ind. t test	0.11
Age group	19–30	39 (30.5%)	10 (16.7%)	Chi-square	0.12
	31–45	68 (53.1%)	40 (66.7%)		
	> 46	21 (16.4%)	10 (16.7%)		
History of high BP	No	97 (75.8%)	48 (80.0%)	Chi-square	0.52
	Yes	31 (24.2%)	12 (20.0%)		
History of Diabetes	No	111 (86.7%)	51 (85.0%)	Chi-square	0.75
	Yes	17 (13.3%)	9 (15.0%)		
Marital status	Single	26 (20.5%)	9 (15.0%)	Fisher’s exact	0.22
	Married	72 (56.7%)	42 (70.0%)		
	Divorced	29 (22.8%)	9 (15.0%)		
Highest level of education	Never studied	11 (8.7%)	6 (10.0%)	Fisher’s exact	0.17
	Preprimary	7 (5.5%)	1 (1.7%)		
	Primary	61 (48.0%)	19 (31.7%)		
	Secondary	34 (26.8%)	25 (41.7%)		
	Tertiary	8 (6.3%)	6 (10.0%)		
	University	6 (4.7%)	3 (5.0%)		
HIV status	Negative	71 (55.5%)	31 (51.7%)	Fisher’s exact	0.38
	Positive	57 (44.5%)	28 (46.7%)		
	Unknown	0 (0.0%)	1 (1.7%)		
HIV Viral Load (copies/ml)		12.6 (± 37.44)	72.07 (± 228.79)	Ind. t test	0.036
CD4 count cells/UL		609.58 (± 277.19)	357.29 (± 438.95)	Ind. t test	0.14
Smoking	No	123 (96.1%)	56 (94.9%)	Fisher’s exact	0.71
	Yes	5 (3.9%)	3 (5.1%)		
Contraceptive use	No	56 (45.5%)	36 (60.0%)	Chi-square	0.066
	Yes	67 (54.5%)	24 (40.0%)		
Age at sexual debut		18.31 (± 3.16)	19.89 (± 3.37)	Ind. t test	0.002
Region of residence	Mbarara city	125 (97.7%)	58 (96.7%)	Fisher’s exact	0.39
	Central Uganda	3 (2.3%)	1 (1.7%)		
	Rest of Western Uganda	0 (0.0%)	1 (1.7%)		

### Distribution of cytology results across general obesity

From the 60 study participants who had general obesity, 38.3% (23/60) were diagnosed with low grade squamous intraepithelial lesion. However, majority of low grade squamous intraepithelial lesions were diagnosed among non-obese women (45.3%). We observed a weakly significant distribution of cytology results across general obesity. Table 3.

**Table 3:** Distribution of cytology results based on general obesity

		No General Obesity	General Obesity	Test	p-value
		N = 128	N = 60		
Cytology result	ASCUS	1 (0.8%)	0 (0.0%)	Fisher’s exact	0.095
	HSIL	11 (8.6%)	1 (1.7%)		
	LSIL	58 (45.3%)	23 (38.3%)		
	NILM	58 (45.3%)	36 (60.0%)		

### Factors associated with general obesity among study participants

We performed logistic regression analysis, first at bivariate and then multivariate. We found that residence in Mbarara city and being in the age groups of 31–45 and 45 ≤ were significantly associated with general obesity among the study participants. Table 4.

**Table 4:** Factors associated with general obesity among study participants

Variable		Bivariate analysis				Multivariate analysis		
		OR	95% CI	P value		AOR	95% CI	P value
Marital status	Single	1						
	Married	1.748	0.761–4.014	0.188		1.749	0.682–4.485	0.245
	Divorced	0.938	0.328–2.678	0.904		0.895	0.277–2.894	0.853
Highest level of education	Never studied	1						
	Preprimary	0.222	0.023–2.187	0.197		0.229	0.021–2.509	0.228
	Primary	0.567	0.188–1.712	0.314		0.690	0.214–2.223	0.535
	Secondary	1.250	0.416–3.755	0.691		1.724	0.517–5.755	0.376
	Tertiary	1.556	0.387–6.254	0.534		1.516	0.327–7.035	0.595
	University	0.857	0.161–4.554	0.856		1.174	0.19–7.239	0.863
Contraceptive use	No	1						
	Yes	0.546	0.373–1.261	0.225		0.576	0.286–1.159	0.122
Region of residence	Rest of Western Uganda	1						
	Mbarara city	1.661	0.904–3.050	0.102		2.156	1.085–4.282	0.028*
	Central Uganda	1.539	0.134–17.667	0.729		2.195	0.148–32.586	0.568
Age group	19–30	1						
	31–45	2.421	1.107–5.301	0.027		3.913	1.577–9.705	0.003*
	≥ 46	1.971	0.698–5.566	0.200		3.376	1.022–11.157	0.046*

OR: Odds Ratio; AOR: Adjusted Odds Ratio

Multivariate logistic regression analysis was done, adjusting for: Marital status, Highest level of education, Contraceptive use, Region of residence and Age group.

### Association between obesity and grades of cervical intraepithelial neoplasia

Table 5 shows results from logistic regression analysis putting into account other demographic factors. General obesity did not show any statistically significant association with cervical intraepithelial neoplasia. University education and contraceptive usage were among the other factors that were significantly associated with cervical intraepithelial neoplasia.

**Table 5:** Logistic regression analysis showing association between general obesity and other factors and Cervical Intraepithelial Neoplasia

	OR		p-value	95% confidence interval			AOR	p-value	95% confidence interval	
				lower limit	upper limit				lower limit	upper limit
Obesity										
Yes	0.543		0.055	0.291	1.013		0.545	0.099	0.265	1.120
**Highest level of education**										
Preprimary	0.720	0.472		0.317	12.009		2.082	0.474	0.280	15.499
Primary	4.301	0.018		1.288	14.366		3.373	0.058	0.958	11.870
Secondary	2.390	0.166		0.696	8.208		2.252	0.225	0.606	8.366
Tertiary	5.850	0.027		1.222	27.994		3.857	0.123	0.695	21.401
University	26.000	0.007		2.451	275.826		27.969	**0.008**	2.371	329.852
**Smoking**										
Yes	7.488	0.062		0.903	62.125		8.126	0.066	0.873	75.635
**Contraceptive use**										
Yes	2.130	0.012		1.179	3.848		2.235	**0.029**	1.087	4.596
**Age group**										
31–45	0.657	0.250		0.322	1.343		0.692	0.411	0.288	1.663
> 46	0.095	0.035		0.011	0.850		0.097	0.060	0.008	1.105

*OR: Odds Ratio; AOR: Adjusted Odds Ratio*

*Multivariate logistic regression analysis was done, adjusting for*: Smoking, *Highest level of education, Contra*ceptive use, *and Age group.*

## Discussion

Obesity is one of the five components of metabolic syndrome that presents with unhealthy amounts and or distribution of fat in one’s body [37] and it is known to increase one’s chances of developing heart diseases and other non-communicable diseases including cervical cancer [38]. There is a reported increased mortality from cervical cancer among obese women [27]. Cervical cancer develops quite slowly, through precancerous stages called cervical intraepithelial neoplasia (CIN) [39]. Therefore, early detection of metabolic syndrome components in CIN is of great significance for prevention of heart and other related cardiovascular diseases.

To our knowledge, there is no other study that has reported prevalence and association between obesity and CIN in a Ugandan population. This study showed that the prevalence of general obesity was 26% among women with cervical intraepithelial neoplasia. General obesity, though not statistically significant, was more prevalent in participants with low grade squamous intraepithelial lesions (38.3%) compared with other grades but this observation was not statistically significant. Previous studies have equally found a similar finding [40]. The possible explanation for this high prevalence of obesity in CIN is that fact that there is an increased serum expression of sex hormones which themselves are thought to increase risk of cancer [41, 42].

This shows that much as many low grade squamous intraepithelial lesions regress on their own, there is an increased risk of coronary heart disease, diabetes, stroke and other cardiovascular diseases [38, 43–45] among patients with low grade squamous intraepithelial lesion due to obesity.

This study also showed that residing in Mbarara city and being in the age brackets of 31–45 and ≥ 46 were significantly associated with obesity among the study participants. It is important to note that the study site, Mbarara Regional Referral Hospital is located in Mbarara city, an urban city in South western Uganda with a relatively high socioeconomic status, and it contributed a big fraction of study participants. This could partly explain the high prevalence of obesity and its association with region of residence, since there is an association between obesity and a high socioeconomic status [46–48]. Similar findings about age and obesity were reported in a Nigerian population [49].

We did not find any association between obesity and cervical precancerous lesions. There have been a lot of inconsistencies in reported studies on association between obesity and CIN or cervical cancer [21, 25–28, 42, 50, 51]. However, our observation is consistent with some previous studies which have shown that obesity is not associated with cervical cancer or its precancerous lesions [21, 25–29, 42, 50]. For instance, a systematic review by Poorolajal et al. [21] only revealed a weak association between obesity and cervical cancer. A lower risk of CIN and higher risk of cervical cancer was reported among obese women [52] and this observation was attributed to a low detection rate of CIN among obese women [53, 54]. Similarly, a study among Thai women showed an increased prevalence of abnormal Pap smear results among obese women though not statistically significant [40].

To the contrary, an association between increased body mass index and a reduce risk of precancerous lesions was reported in a prospective cohort of Koreans [51]. Specifically, from a cross sectional study conducted in a Nigerian population, there was a reported association between obesity and epithelial cell abnormalities [49].

### Strengths and limitations

We acknowledge that our study did not have a big sample size and this might have hindered observation of associations between variables. We also did not capture information on participant HPV vaccination status. Our current guidelines dictate vaccination of only young girls (9–14 years). In any case, we did not expect adults in our study to have been vaccinated considering the fact that HPV vaccination is a new intervention that has just been rolled out.

## Conclusion

There was a high prevalence of obesity among women with CIN especially low grade squamous intraepithelial lesions. We did not observe any statistically significant association between obesity and CIN. Factors associated with obesity among our study participants included residence in Mbarara city and being in the age groups of 31–45 and ≥ 45 years. This emphasises the need to rethink management of CIN and cervical cancer to control other non-communicable diseases that could arise due to obesity. We recommend prospective cohort studies to further understand obesity and CIN as well as cervical cancer.

## Data Availability

All data from which this article was generated is available from the corresponding author upon meaningful request.

## Abbreviations

CIN: Cervical Intraepithelial neoplasia
VIA: Visual Inspection with acetic acid
LEEP: Loop Electrosurgical Excision Procedure
PAP: Papanicolaou
BMI: Body Mass Index
LSIL: Low Grade Squamous Intraepithelial Lesion
STATA: Statistical Software for Data Science
AOR: Adjusted Odds Ratio
OR: Odds Ratio
CI: Confidence Interval
SD: Standard Deviation
HPV: Human Papilloma Virus
CVD: Cardiovascular Disease
HIV: Human Immunodeficiency Virus
